# Ethoxylation-Dependent Self-Assembly Behavior and Enhanced Oil Recovery Performance of P(AA-AAEO_n_) Amphiphilic Copolymers

**DOI:** 10.3390/polym17172269

**Published:** 2025-08-22

**Authors:** Xiqiu Wang, Shixiu Wang, Kaitao Xin, Guangyu Wang, Liping Pan, Yannan Ji, Weiping Lu

**Affiliations:** 1 School of Electrical and Energy Engineering, Nantong Institute of Technology, Nantong 226002, China; 20246143@ntit.edu.cn (X.W.); 20246336@ntit.edu.cn (G.W.); panlp@ntit.edu.cn (L.P.); 15051243669@163.com (Y.J.); luweiping2610@126.com (W.L.); 2 College of Chemistry and Chemical Engineering, China University of Petroleum (East China), Qingdao 266580, China; cbiotechnology@163.com

**Keywords:** amphiphilic copolymer, hydrophobic association, molecular self-assembly, interfacial activity, enhanced oil recovery

## Abstract

This study examined a novel ethoxy-segment-regulated hydrophobic associative amphiphilic copolymer, P(AA-AAEO_n_), and systematically evaluated its solution self-assembly behavior and enhanced oil recovery (EOR) performance. The influence of ethylene oxide (EO) chain length and polymer concentration on particle size distribution and aggregation morphology was analyzed using dynamic light scattering (DLS). The results revealed a concentration-dependent transition from intramolecular to intermolecular association, accompanied by a characteristic decrease followed by an increase in hydrodynamic diameter. At a fixed AA:AAEO_n_ molar ratio (400:1), increasing EO segment length increased aggregate size and improved colloidal stability. Viscometric analysis showed that longer EO chains markedly increased molecular chain flexibility and solution viscosity. Interfacial tension measurements demonstrated superior interfacial activity of P(AA-AAEO_n_) compared to polyacrylic acid (PAA), and longer EO chains further reduced oil–water interfacial tension. Emulsification tests verified its strong ability to emulsify crude oil. Sandpack flooding experiments and micromodel studies demonstrated effective conformance control and high displacement efficiency, achieving up to 30.65% incremental oil recovery. These findings offered essential insights for designing hydrophobic associative polymers with tunable interfacial properties for EOR applications.

## 1. Introduction

Hydrophobically associating water-soluble polymers (HAWSPs) represent an important class of functional materials that combines the viscoelastic properties of polymer solutions with the interfacial activity of surfactants by incorporating hydrophobic moieties into hydrophilic backbones [1,2,3]. Recent advances in HAWSP design have focused on optimizing molecular architecture to control self-assembly behavior in aqueous environments. For instance, Zhang et al. [4] developed lysine-derived amphiphilic cyclic polymers with tunable hydrophilic/hydrophobic ratios, which enabled stable micelle formation for enhanced drug delivery. Liu et al. [5] employed polymerization-induced self-assembly (PISA) coupled with coarse-grained simulations and demonstrated that monomer reactivity governed the assembly pathways and nanostructures of gradient copolymers. Guo et al. [6] designed a dual-hydrophobic-group amphiphilic copolymer (PAALB) that exhibited high interfacial activity and significant viscosity reduction at high oil–water ratios, highlighting its potential for enhanced oil recovery (EOR) in challenging reservoir conditions. In addition, recent studies have explored the interplay between hydrophobic association and polymer topology, showing that branched or star-shaped architectures could significantly enhance solution viscoelasticity and interfacial performance compared to linear analogs [7,8,9,10]. Advanced characterization techniques, including small-angle neutron scattering (SANS) and rheo-optical methods, have provided direct insights into micellar network formation, aggregation dynamics, and response to external stimuli, further deepening the understanding of HAWSP solution behavior [11,12,13].

Despite these advances, existing HAWSP systems have predominantly relied on ionic structures or long-chain alkyl hydrophobic monomers, which left a critical gap in understanding the associative mechanisms of non-ionic units in the absence of electrostatic interactions [14,15,16]. The role of non-ionic segments in modulating solution microstructure and interfacial properties was poorly understood [17,18,19,20], with most studies limited to basic rheological and surface tension measurements. This lack of mechanistic insight into structural evolution and interfacial dynamics under reservoir conditions has hindered the practical deployment of HAWSPs in field applications [21,22,23].

Polyoxyethylene ether (AEO_n_) non-ionic monomers are considered to offer a promising solution due to their tunable ethylene oxide (EO) chain length, excellent aqueous solubility, and biocompatibility [24,25,26,27]. Previous investigations on AEO_n_-based polymers have shown that EO chain length critically influenced micelle formation, hydration layer structure, and interfacial packing density, suggesting that systematic tuning of EO segments could provide a versatile strategy for controlling non-ionic hydrophobic associations [28,29]. Moreover, the combination of AEO_n_ units with other functional monomers was reported to enhance emulsification stability and reduce interfacial tension more efficiently than conventional long-chain alkyl systems, pointing toward their potential in advanced EOR formulations [30,31].

By incorporating AEO_n_ units into polymer backbones, non-ionic associative domains were engineered in HAWSPs, thereby addressing the limitations of conventional systems in terms of solution stability and structural control while enhancing key functionalities such as viscosity modification, emulsification, and interfacial activity. In this study, the P(AA-AAEO_n_) copolymer system was systematically investigated in order to elucidate how EO segment length and polymer concentration governed solution behavior and interfacial properties. Through comprehensive characterization of hydrodynamic diameter evolution, intrinsic viscosity, oil–water interfacial tension, and emulsification stability, coupled with performance evaluation under simulated reservoir conditions, structure–property relationships were established to support the development of non-ionic HAWSPs for EOR applications.

## 2. Experimental

### 2.1. Materials

Acrylic acid (AA), cyclohexane, p-toluenesulfonic acid (PTSA), p-phenylene ether, and ammonium persulfate (APS) were obtained from Sinopharm Chemical Reagents Co., Ltd. (Shanghai, China). All chemicals were of analytical grade (AR) and were used directly without further purification. The non-ionic amphiphilic monomers AEO_n_, with polyethylene oxide (PEO) chain lengths of *n* = 5, 10, and 15 (denoted as AEO_5_, AEO_10_, and AEO_15_, respectively), were synthesized according to previously reported methods [32].

### 2.2. Dynamic Light Scattering Measurements

**Figure 1:**
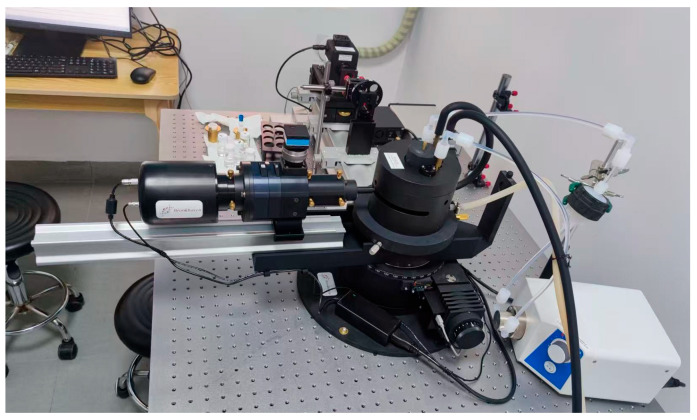
BI-200SM wide-angle dynamic/static light scattering instrument.

Dynamic light scattering (DLS) measurements were conducted at various concentrations using a BI-200SM wide-angle dynamic/static light scattering instrument (Brookhaven Instruments, Holtsville, NY, USA), as shown in Figure 1. The measurements were carried out at a fixed scattering angle of 90°, with a laser wavelength of 532 nm and a pinhole aperture of 100 μm. Following calibration, particle size distributions were derived using the non-negative least squares (NNLS) algorithm, with scattered light intensity maintained below 500 Kcps. The influence of concentration and monomer composition on micelle size was systematically investigated for the three hydrophobically associating amphiphilic copolymers: P(AA-AAEO_5_), P(AA-AAEO_10_), and P(AA-AAEO_15_).

### 2.3. Determination of Intrinsic Viscosity and Viscosity-Average Molecular Weight

The intrinsic viscosity ([*η*]) and viscosity-average molecular weight (*M_η_*) of the hydrophobically associating polymer P(AA-AAEO_n_) were determined using the dilution method with an Ubbelohde viscometer, following the polyacrylamide molecular weight determination standard (GB12005.1-1989) [33] and the hydrophobic associative polymer protocol reported by Tam, K.C. et al. [34]. Both parameters were determined by the one-point method [35]. The experimental procedure was as follows:

(1) Sample Preparation: The P(AA-AEO_n_) sample was dried at 100 °C until a constant weight was achieved. The Ubbelohde viscometer was thoroughly cleaned and dried to prevent contamination.
(2) Solution Preparation: Precisely 0.20–0.30 g of the dried sample was dissolved in 1 M NaCl solution (to maintain constant ionic strength), transferred to a 100 mL volumetric flask, diluted to the mark, and thoroughly mixed. The resulting solution was equilibrated at 25.0 ± 0.1 °C.
(3) Solvent Baseline: The Ubbelohde viscometer was vertically suspended in a thermostated water bath maintained at 25.0 ± 0.1 °C. Approximately 10 mL of 1 M NaCl solution was loaded, and the system was equilibrated for 10 min. The efflux time was measured in triplicate, with a deviation of less than 0.2 s, and the average solvent flow time (*t*_0_) was recorded.
(4) Polymer Measurement: The NaCl solution was replaced by the P(AA-AEO_n_) sample solution, and measurements were repeated to determine the average polymer solution flow time (*t*).

### 2.4. Interfacial Activity Test

Interfacial tension was measured using the rotating drop method, a technique suitable for low-interfacial-tension systems and widely applied in enhanced oil recovery studies [36]. Prior to measurement, the capillary tube was sequentially rinsed with petroleum ether, ethanol, and deionized water, and then dried. The test solution was introduced into the tube, followed by the careful injection of a 2–3 μL droplet of crude oil using a microsyringe. The tube was sealed and mounted in the rotating chamber of the interfacial tensiometer. Measurements were performed at a rotational speed of 5000 rpm over the temperature range of 30–60 °C in 10 °C increments. For each test, interfacial tension was recorded as a function of rotation time until equilibrium was reached. All measurements were repeated in triplicate, and the average equilibrium interfacial tension values were reported.

### 2.5. Emulsification Test

The emulsification performance of the hydrophobically associating amphiphilic copolymer solutions was evaluated according to previously reported protocols [37,38]. Polymer solutions were equilibrated at 45 °C for 15 min in a thermostatic water bath. Crude oil was then added to the aqueous phase under controlled oil-to-water ratios, followed by high-shear emulsification at 1000 rpm using a laboratory emulsifier. The effects of polymer concentration, salinity, and oil–water ratio on emulsification behavior were systematically investigated for three different systems. Emulsion morphology was observed via optical microscopy, while emulsion stability was assessed by monitoring phase separation over time at 45 °C. The emulsification capacity was expressed in terms of both droplet size distribution and stability index.

### 2.6. Simulated Glass Simulation Water-Blocking Experiment

The glass micromodel water-blocking experiment involved using a physical model to simulate fluid flow and water-blocking behavior under reservoir conditions [39,40,41]. The effectiveness of the oil displacement fluid was evaluated via microscopic observation. A 2 × 2 cm two-dimensional glass micromodel with an average pore diameter of 20 μm was employed to observe the oil–water distribution during displacement by P(AA-AAEO_n_). The solution was injected at a constant rate of 0.003 mL/min using a micro-pump. After saturating the micromodel with simulated oil, water was injected until no further oil was produced. The polymer solution was then introduced at the same rate until oil recovery ceased. Residual oil distribution was observed and analyzed to evaluate the plugging performance of the polymer system. An optical microscope equipped with a digital camera continuously recorded the oil–water distribution during injection to enhance experimental clarity. Additionally, polymer solution viscosity was measured prior to injection using a rotational rheometer to correlate rheological properties with plugging performance.

### 2.7. Sand-Filled Tube Experiment

The sand-packed tube displacement test simulated the injection process in actual oilfield development, using quartz sand to mimic the pore structure of reservoir rocks and enabling the study of oil–water separation and oil displacement efficiency in porous media [42]. The experiment was conducted at 50 °C to evaluate the oil displacement performance of P(AA-AAEO_n_). The sand-packed tube was filled with quartz sand, with a measured permeability during water injection of 3.06 μm^2^. After saturating the tube with crude oil, the system was aged for 24 h. Water flooding was initiated at 0.5 mL/min until the water content of the produced fluid exceeded 98%. Polymer flooding was conducted using 0.7 wt% P(AA-AAEO_n_) solution at the same injection rate, continuing until the effluent water content again exceeded 98%. Water flooding was resumed to assess post-polymer oil recovery. The effluent was periodically collected in graduated cylinders, and inlet and outlet pressures were continuously monitored using digital pressure sensors to track flow resistance and plugging behavior, providing detailed experimental information. The sand tube and polymer solution were preheated to 50 °C to maintain a consistent temperature, and polymer viscosity and density were measured prior to injection to analyze their effects on oil displacement efficiency.

## 3. Results and Discussion

### 3.1. Hydrodynamic Size Determination

**Figure 2:**
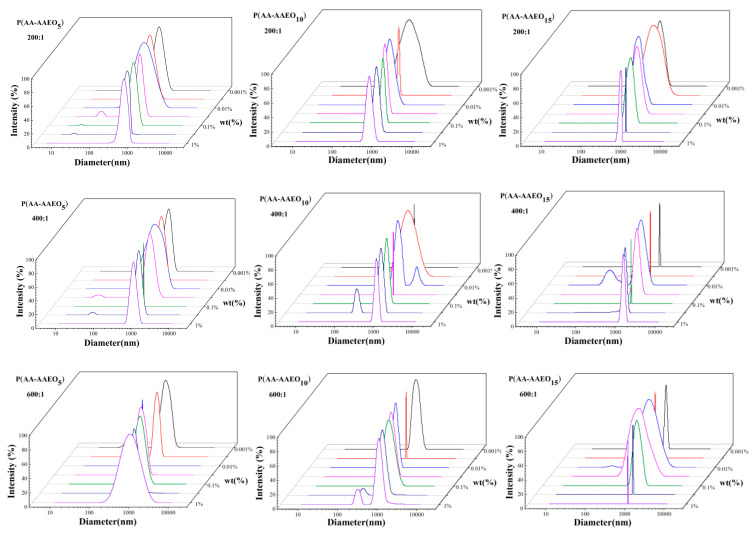

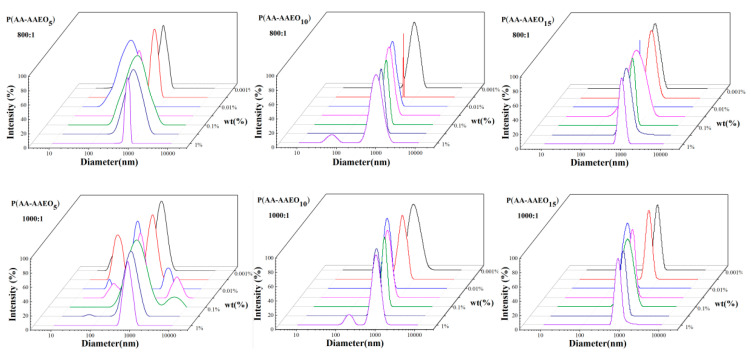
Hydrodynamic size distribution of P(AA-AAEO_n_) at various concentrations and AEO_n_:AA molar ratios.

The hydrodynamic size distribution of P(AA-AAEO_n_) in aqueous solution was systematically investigated over a concentration range of 0.001–1 wt%. Figure 2 shows a concentration-dependent bimodal shift in the size distribution, with the dominant peak initially moving toward smaller hydrodynamic diameters, followed by a shift to larger sizes. This non-monotonic behavior indicates an initial decrease followed by an increase in the apparent hydrodynamic diameter of P(AA-AAEO_n_) assemblies. At low concentrations (<0.01 wt%), an extended chain conformation predominated due to weak intermolecular interactions, enabling polymer chains to retain their maximum hydrodynamic volume. As the concentration increased (0.01–0.1 wt%), polymer chains underwent contraction driven by intramolecular hydrophobic interactions. Beyond 0.1 wt%, the system transitioned to intermolecular associations, forming larger supramolecular aggregates via hydrophobic domain interactions [43].

The hydrodynamic size evolution of P(AA-AAEO_n_) assemblies displays a non-monotonic dependence on monomer composition. A gradual increase in hydrodynamic diameter is observed as the AEO_n_:AA molar ratio decreases from 1000:1 to 400:1. However, a further reduction to a molar ratio of 200:1 results in an unexpected decrease in assembly size. Consequently, the largest assembly size is observed at an optimal molar ratio of 400:1. This trend is attributed to enhanced incorporation of AEOn segments into copolymer chains at moderate ratios, facilitating chain elongation and molecular expansion. At lower ratios, excessive hydrophobic segment density promotes intramolecular associations, thereby driving chain compaction and causing size reduction. This transition from chain extension to hydrophobic collapse explains the observed maximum size at intermediate compositions [44,45].

### 3.2. Intrinsic Viscosity and Viscosity-Average Molecular Weight

The intrinsic viscosity [*η*] of a polymer provides insight into the strength of intermolecular interactions and the influence of molecular conformation on flow resistance at a given temperature. Typically, polymers with higher molecular weights exhibit larger intrinsic viscosities due to increased hydrodynamic volume and chain entanglement.

The intrinsic viscosity [*η*] and viscosity-average molecular weight (*M_n_*) of the P(AA-AAEO_n_) copolymer (with an AEO_n_:AA monomer molar ratio of 400:1) were determined using an Ubbelohde viscometer. The following equations were applied for the calculation:

(1) $$\eta_{r}=\frac{t}{t_{0}}$$

(2) $$\left[\eta \right]=\frac{\sqrt{\frac{2-\ln3}{\eta_{r}-\ln\eta -1}}\left(\eta_{r}-1\right)-2\sqrt{\frac{\eta_{r}-\ln\eta -1}{2-\ln3}}}{c\left(\sqrt{\frac{2-\ln3}{\eta_{r}-\ln\eta -1}}-1\right)}$$

(3) $$\left[\eta \right]=KM_{n}^{\alpha }$$

(4) $$M_{n}={\left(\frac{\left[\eta \right]}{K}\right)}^{\frac{1}{\alpha }}$$

where

*ƞ_r_*—relative viscosity;

[*ƞ*]—characteristic viscosity number;

*M_n_*—viscosity-average molecular weight of the polymer;

*t*—efflux time of the polymer solution, s;

*t*_0_—efflux time of the reference NaCl solution, s;

*c*—polymer concentration, g/mL.

*K* and *α* are constants, referenced from the Mark–Houwink equation for hydrophobic associative polymers calibrated by Ohshima, A. et al. [46]. Here, *K* is taken as 0.182 and *α* as 0.586 to determine the molecular weight of the polymer.

As summarized in Table 1, both the intrinsic viscosity and viscosity-average molecular weight of the P(AA-AAEO_n_) copolymers increased with the number of EO units incorporated. This trend is attributed to the significant influence of EO segments on polymer conformation and hydrophilicity. On one hand, EO segments possess high flexibility and polarity, promoting chain expansion in aqueous environments and increasing the hydrodynamic volume, which enhances the intrinsic viscosity. On the other hand, the strong solvation ability and steric bulk of EO units suppress excessive intra- and intermolecular coiling or aggregation, thereby stabilizing more extended chain conformations and favoring the formation of larger supramolecular complexes. Therefore, increasing the EO content enhances both the molecular extensibility and the effective intermolecular interactions, resulting in a concurrent rise in intrinsic viscosity and *M_n_*.

The apparent viscosity of aqueous solutions was measured at 25 °C over a shear rate range of 0.1–1000 s^−1^. The flow behavior was analyzed using the power-law model to obtain the consistency index *K* and flow behavior index *n* (Table 2), where *K* reflects the viscosity magnitude and n indicates deviation from Newtonian behavior (*n* = 1 for Newtonian, *n* < 1 for pseudoplastic, *n* > 1 for dilatant). All samples exhibited pseudoplastic behavior (*n* < 1), and increasing EO chain length slightly increased *n*, indicating enhanced chain flexibility and a reduced shear-thinning tendency. These results establish a clear structure–property correlation: higher intrinsic viscosity and molecular weight, induced by increased EO content, are associated with higher consistency indices and reduced shear-thinning, reflecting the combined influence of polymer conformation and intermolecular interactions on macroscopic rheological behavior.

### 3.3. Interfacial Activity

**Figure 3:**
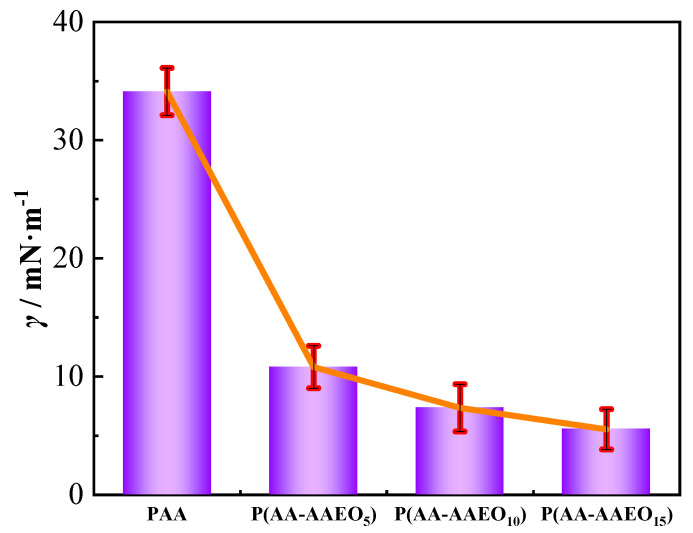
Interfacial activity.

Interfacial activity is a key parameter for evaluating a polymer’s capability to adsorb at the oil–water interface and modulate interfacial behavior, and is typically assessed by measuring the reduction in interfacial tension between the oil and aqueous phases [47]. To systematically assess the interfacial performance of the synthesized P(AA-AAEO_n_) copolymers, the oil–water interfacial tension was measured under controlled conditions: 1 wt% polymer concentration and a fixed AA:AEO_n_ molar ratio of 400:1. Pure poly(acrylic acid) (PAA) was used as a reference. As shown in Figure 3, all P(AA-AAEO_n_) copolymers exhibited significantly enhanced interfacial activity compared to PAA, effectively lowering the oil–water interfacial tension. Notably, increasing the number of EO units in the AEO_n_ segments resulted in a further reduction in interfacial tension, indicating a strong dependence of interfacial behavior on EO segment length.

The enhanced interfacial activity of P(AA-AAEO_n_) copolymers is primarily attributed to the incorporation of non-ionic, hydrophilic AEO_n_ segments with intrinsic surface-active properties [48]. These segments facilitate rapid migration of the polymer chains to the oil–water interface and promote stable adsorption. The EO side chains enable effective amphiphilic alignment at the interface, with hydrophilic polyether backbones remaining solvated in the aqueous phase, while hydrophobic moieties interact favorably with the oil phase [49]. Notably, copolymers containing longer EO chains, such as P(AA-AAEO_15_), display superior interfacial orientation behavior due to increased molecular flexibility and steric volume. These structural features favor the formation of a compact, densely packed adsorption layer at the interface, enhancing interfacial film stability and providing stronger barrier properties against coalescence or emulsification. Consequently, the pronounced reduction in oil–water interfacial tension observed for P(AA-AAEO_15_) correlates with improved emulsification stability and indicates potential for enhanced oil displacement efficiency in aqueous-phase flooding applications [50].

### 3.4. Emulsifying Properties

Hydrophobically associative amphiphilic copolymers exhibit intrinsic and efficient emulsifying capabilities due to the presence of both hydrophilic and hydrophobic segments within their molecular structure. The hydrophilic moieties preferentially interact with the aqueous phase, forming a stable hydration shell that provides both steric and electrostatic stabilization to dispersed oil droplets, thereby inhibiting droplet coalescence and enhancing dispersion stability. Simultaneously, the hydrophobic segments tend to partition into the oil phase or adsorb strongly onto the oil–water interface, anchoring the copolymer to the droplet surface and further suppressing coalescence through interfacial anchoring and steric hindrance. These amphiphilic interactions collectively drive spontaneous self-assembly of the copolymer chains at the oil–water interface, minimizing interfacial free energy and forming a robust, stabilized interfacial film. As a result, the copolymers function effectively as polymeric emulsifiers, significantly reducing interfacial tension and facilitating the formation of thermodynamically stable emulsions, which is critical for enhancing oil displacement efficiency, particularly under harsh reservoir conditions.

**Figure 4:**
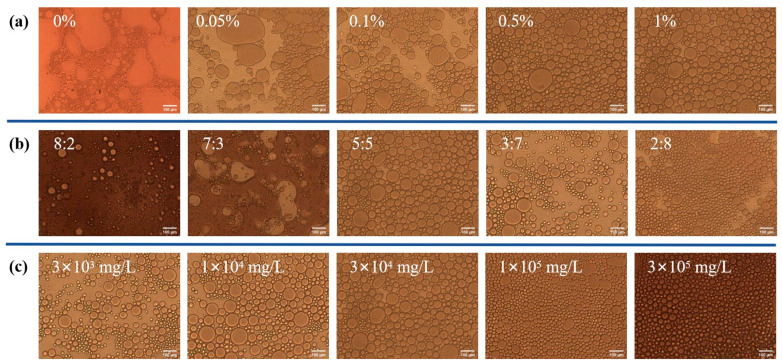
Particle size distribution of P(AA-AAEO_n_) under different concentrations, oil–water ratios, and salinity conditions: (**a**) different polymer concentrations; (**b**) different oil–water ratios; (**c**) different salinities.

At a fixed NaCl concentration of 3 × 10^4^ mg/L, the addition of P(AA-AAEO_15_) significantly modifies the emulsion behavior. In the absence of polymer, the system displays an unstable and polydisperse oil-in-water emulsion, as shown in Figure 4a. As the polymer concentration increases from 0 wt% to 1 wt%, the emulsion gradually transforms into a stable water-in-oil type, with a narrower droplet size distribution and reduced mean droplet diameter. This improvement is attributed to the enhanced adsorption of amphiphilic copolymer chains at the oil–water interface, which lowers interfacial tension and promotes the breakup of larger oil droplets into finer ones, thereby increasing emulsion stability.

At a constant polymer concentration of 1 wt% and a NaCl concentration of 3 × 10^4^ mg/L, the influence of different oil-to-water ratios (8:2, 7:3, 5:5, 3:7, and 2:8) on emulsification behavior was assessed, as shown in Figure 4b. With decreasing oil content, the system undergoes a phase inversion from an oil-in-water to a water-in-oil emulsion. The inversion point occurs at an oil-to-water ratio of approximately 7:3. When the continuous phase is water (low viscosity), dispersed oil droplets are more effectively stabilized, resulting in transparent emulsions with reduced flow resistance.

Under fixed conditions of polymer concentration (1 wt%) and oil–water ratio (5:5), the effect of salinity on emulsion characteristics was investigated, as shown in Figure 4c. Increasing salinity leads to enhanced emulsion uniformity and smaller droplet sizes. The presence of salts increases the solution polarity and promotes hydrophobic interactions among polymer chains. Consequently, a denser and more compact interfacial film is formed around the droplets, improving the emulsion’s structural integrity and stability under high-salinity environments.

### 3.5. Micro-Scale Simulation of Oil Displacement

**Figure 5:**
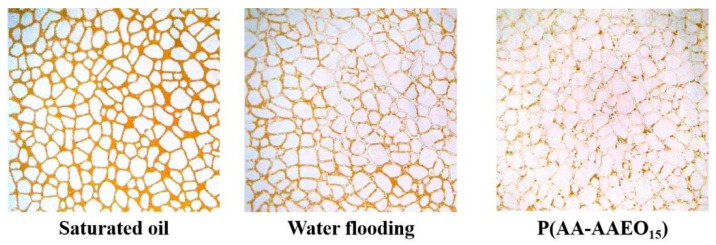
Micro-scale simulation of displacement.

To investigate the mechanisms of oil displacement and the distribution of residual oil at the pore scale, microfluidic visualization experiments were conducted using a flat glass-etched model saturated with crude oil. The displacement behaviors of water and a 1 wt% solution of the hydrophobic associative amphiphilic copolymer P(AA-AAEO_15_) (monomer molar ratio AA:AEO_n_ = 400:1) were compared, and the results are presented in Figure 5.

After conventional water flooding, a significant amount of residual oil remained within the pore network. This inefficiency was primarily attributed to the pronounced mobility contrast between water and oil, which led to low sweep efficiency and the formation of dominant flow channels. The injected water rapidly advanced along these preferential paths, bypassing large regions of the oil-bearing zone and leaving unswept residual oil. The low viscosity of the injected water resulted in insufficient displacement pressure and incomplete oil mobilization. In contrast, after subsequent injection of the gel-breaking solution of P(AA-AAEO_15_), a substantial reduction in residual oil saturation was observed. The amphiphilic copolymer significantly improved displacement efficiency by increasing the viscosity of the displacing phase and expanding the sweep volume. Additionally, the polymer chains interacted with the pore structure and crude oil droplets, promoting the mobilization and entrainment of previously trapped oil clusters. These results demonstrated that polymer flooding following water flooding could effectively recover additional residual oil, providing a promising strategy for enhanced oil recovery at the micro-scale.

### 3.6. Sand-Pack Oil Displacement Experiment

**Figure 6:**
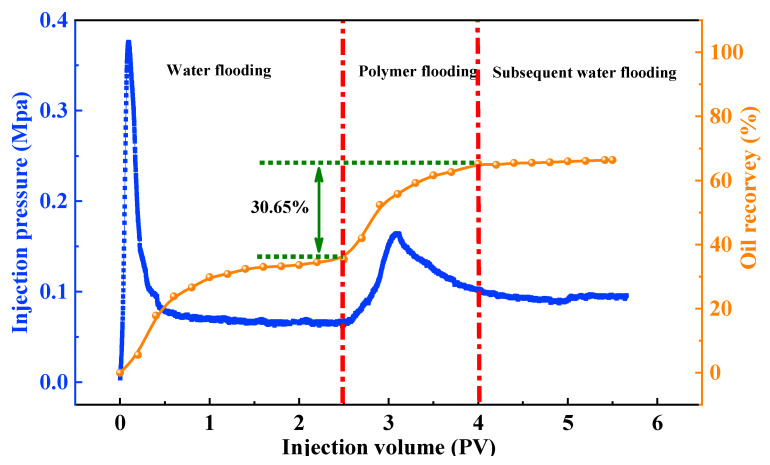
Oil recovery curve for P(AA-AAEO_15_) flooding in a sand-pack system.

The oil displacement performance of the amphiphilic copolymer P(AA-AAEO_15_) (monomer molar ratio AA:AEO_n_ = 400:1) at a concentration of 0.7 wt% was evaluated using a sand-packed column. The displacement process was divided into three stages, namely, water flooding, polymer flooding, and subsequent water flooding, as illustrated in Figure 6.

During the initial water flooding stage, the injection pressure differential rose sharply to 0.37 MPa, followed by a rapid decline to 0.08 MPa due to the formation of preferential flow channels after water breakthrough. After injecting 2.5 pore volumes (PV) of water, the water cut increased rapidly to over 98%, and the oil recovery factor reached 35.07%, indicating limited sweep efficiency caused by viscous fingering and channeling. In the polymer flooding stage, the pressure differential increased to 0.16 MPa, indicating improved flow resistance and better conformance control. Upon injection of 1.5 PV of the P(AA-AAEO_15_) solution, the water cut remained above 98%, and the oil recovery factor increased by an additional 30.65%, demonstrating the copolymer’s excellent oil displacement capability. During the subsequent water flooding stage, the pressure differential and water cut remained stable, with negligible further increase in oil recovery. After injecting a total of 1.5 PV of follow-up water, the final cumulative recovery reached 65.72%.

These results confirm that P(AA-AAEO_15_) exhibits excellent oil displacement performance, which can be attributed to the synergistic effects of the copolymer’s functional groups. The carboxyl groups in the acrylic acid backbone enhance molecular extensibility, enabling the polymer chains to penetrate low-permeability regions and unswept zones. Simultaneously, the polyethylene oxide (EO) segments in the AEO_n_ side chains display strong hydrophilicity and interfacial activity. These EO segments preferentially adsorb at the oil–water interface and align in an ordered manner, resulting in reduced interfacial tension, improved wettability alteration, and efficient desorption of residual oil films adhered to rock surfaces. This combination facilitates the mobilization and displacement of trapped oil from pore throats, thereby significantly enhancing the overall oil recovery efficiency [51,52].

## 4. Conclusions

In this study, a series of hydrophobically associative amphiphilic copolymers, P(AA-AAEO_n_), were successfully synthesized by incorporating non-ionic polyoxyethylene ether (AEO_n_) units. The effects of EO chain length on the polymers’ solution self-association behavior, interfacial regulation properties, and oil displacement performance were systematically investigated. The results elucidate how structural modulation of non-ionic segments enhances molecular extensibility, interfacial activity, and oil recovery efficiency. Based on these findings, the main conclusions can be summarized as follows:

1. P(AA-AAEO_n_) copolymers exhibited concentration-dependent associative behavior in aqueous solution, transitioning from intramolecular to intermolecular association with increasing concentration.
2. Increasing the EO chain length resulted in higher viscosity-average molecular weight and intrinsic viscosity, indicating improved molecular extensibility and hydrodynamic volume.
3. The copolymers effectively reduced oil–water interfacial tension, suppressed droplet coalescence, and enhanced emulsion stability, demonstrating their strong interfacial functional performance.
4. In microfluidic oil displacement tests, the copolymers markedly decreased residual oil saturation, highlighting their potential for water-blocking mitigation and residual oil mobilization.
5. In sand-packed core flooding experiments, P(AA-AAEO_15_) achieved an additional 30.65% increase in crude oil recovery following conventional water flooding, confirming the practical efficiency of EO-based structural modulation.

In conclusion, this work demonstrates that the rational design of non-ionic EO segments provides an effective strategy to tailor polymer interfacial behavior and enhance oil recovery, offering valuable guidance for the development of advanced EOR agents and their future application in reservoirs.

## Figures and Tables

**Table 1:** [*ƞ*] and *M_n_* of P(AA-AAEO_n_).

	P(AA-AAEO_5_)	P(AA-AAEO_10_)	P(AA-AAEO_15_)
*ƞ_r_*	1.203	1.214	1.217
[*ƞ*] (mL/g)	189.79	199.65	202.32
*M_n_*	1.45 × 10^5^	1.58 × 10^5^	1.62 × 10^5^

**Table 2:** *n* and *K* of P(AA-AAEO_n_).

	*n*	*K*
P(AA-AAEO_5_)	0.72	2.34
P(AA-AAEO_10_)	0.75	2.15
P(AA-AAEO_15_)	0.78	1.98

## Data Availability

All data, models, and code generated or used during the study appear in the submitted article.

## References

[1] Ge S., Shi L., Ye Z., Liu G., Ba Y., Wang X., Yang Y., Chen M., Yuan N., Li P. (2024). Synthesis and Mechanistic Investigation of an Amphiphilic Polymer in Enhancing Extra-Heavy Oil Recovery via Viscosity Reduction. Langmuir.

[2] Qian Q., Xu J., Zhang M., He J., Ni P. (2019). Versatile Construction of Single-Tailed Giant Surfactants with Hydrophobic Poly (ε-caprolactone) Tail and Hydrophilic POSS Head. Polymers.

[3] Iborra A., Díaz G., López D., Giussi J.M., Azzaroni O. (2017). Copolymer based on lauryl methacrylate and poly (ethylene glycol) methyl ether methacrylate as amphiphilic macrosurfactant: Synthesis, characterization and their application as dispersing agent for carbon nanotubes. Eur. Polym. J..

[4] Yan Y., Zhang Q.-L., Tao Y. (2025). Synthesis of lysine-based amphiphilic random copolymers capable of self-assembly in water. Polymer.

[5] Xing J.Y., Li S., Liu B., Liu H., Zhu Y.L. (2025). Polymerization Induced Self-Assembly for Modulating Assembly Pathways and Microstructures of Amphiphilic Gradient Copolymer Nanoparticles. Macromolecules.

[6] Fei D., Guo J., Xiong R., Zhang X., Kang C., Kiyingi W. (2023). Preparation and Performance Evaluation of Amphiphilic Polymers for Enhanced Heavy Oil Recovery. Polymers.

[7] Lee J.H., Goldberg J.M., Fetters L.J., Archer L.A. (2006). Linear Viscoelastic Behavior of Symmetric and Asymmetric Star Polymer Solutions. Macromolecules.

[8] Zhu S., Xu M., Yang S., Huang Z., Wang Y., Zhu Y. (2024). Evaluation of shear resistance of dendritic hydrophobic association polymers. J. Polym. Res..

[9] Wever D.A.Z., Picchioni F., Broekhuis A.A. (2013). Branched polyacrylamides: Synthesis and effect of molecular architecture on solution rheology. Eur. Polym. J..

[10] Gloger D., Mileva D., Albrecht A., Hubner G., Androsch R., Gahleitner M. (2022). Long-Chain Branched Polypropylene: Effects of Chain Architecture, Melt Structure, Shear Modification, and Solution Treatment on Melt Relaxation Dynamics. Macromolecules.

[11] Ren Y., Wei Z., Leng X., Wu T., Bian Y., Li Y. (2016). Relationships between Architectures and Properties of Highly Branched Polymers: The Cases of Amorphous Poly (trimethylene carbonate) and Crystalline Poly (ε-caprolactone). J. Phys. Chem. B.

[12] Mercado R., Martinez R., Rondón M. (2023). Rheological demonstration of micellization changes of internal olefin sulfonate and alcohol alkoxy sulfate commercial surfactants upon interactions with inorganic salts. Results Eng..

[13] Jin Q., Li X., Cai Z., Zhang F., Yadav M.P., Zhang H. (2017). A comparison of corn fiber gum, hydrophobically modified starch, gum arabic and soybean soluble polysaccharide: Interfacial dynamics, viscoelastic response at oil/water interfaces and emulsion stabilization mechanisms. Food Hydrocoll..

[14] Yu Q., Wu X., Li Y., Gao T., Liu S., Hou C., Zheng Z. (2018). Synthesis, Characterization, and Aqueous Properties of an Amphiphilic Terpolymer with a Novel Nonionic Surfmer. Int. J. Polym. Sci..

[15] Zhang G., Yu J. (2021). Effect of commonly used EOR polymers on low concentration surfactant phase behaviors. Fuel.

[16] Khoury J., Seubert A. (2025). Separation of per- and polyfluorinated alkyl substances (PFAS) by means of anion exchange chromatography and study of their retention behavior. J. Chromatogr. A.

[17] Ye Y., Qiu N., Qiu Z., Wang J., He Y., Liu F. (2024). Acetone extraction induced piperazine diffusion reaction for regulating thin film composite nanofiltration membrane. J. Membr. Sci..

[18] Li X., Mei X., Chen M., Li D., Zeng Q., Zhu J., Long S., Huang Y. (2024). Solvent-induced phase separation and Hofmeister effect enhanced strong, tough, and adhesive polyion complex hydrogels. Chem. Eng. J..

[19] Zhang X., Qu Z., Tang Z., Mazhar I. (2024). pH-temperature coupled regulation for promoted nanofluidic osmotic energy conversion. Desalination.

[20] Yoo S., Li Y., Douglas T., Bu W., Dutta P. (2024). Relationship of interface structure to the dynamics of selective lanthanide extraction. Colloids Surf. A Physicochem. Eng. Asp..

[21] Lan K., Zhao D. (2022). Functional Ordered Mesoporous Materials: Present and Future. Nano Lett..

[22] Czelusniak L.E., Mapelli V.P., Guzella M.S., Cabezas-Gómez L., Wagner A.J. (2020). Force approach for the pseudopotential lattice Boltzmann method. Phys. Rev. E.

[23] Pratama R.A., Babadagli T., Temperature E.O., Change P. (2020). and Chemical Additives on Wettability Alteration During Steam Applications in Sands and Carbonates. SPE Reserv. Eval. Eng..

[24] Meier M.A.R., Hu R., Tang B.Z. (2021). Multicomponent Reactions in Polymer Science. Macromol. Rapid Commun..

[25] Cichos F., Thurn-Albrecht T. (2023). Polymers under Multiple Constraints: Restricted and Controlled Molecular Order and Mobility. Macromol. Chem. Phys..

[26] Wang D., Chen J., Li Y., Feng S. (2024). Solid State Chemistry and Inorganic Synthetic Chemistry-Novel Structures and Accurate Syntheses of Inorganic Materials. Small Methods.

[27] Li W., Min J. (2023). Responsive polymer thin films. J. Polym. Sci..

[28] Darvishi S., Senses E. (2023). Interfacial polymer architecture can control nanoparticle dispersion and rheological behavior of nanocomposites. Eur. Polym. J..

[29] Gong L. (2025). Thermal- and Rate-Regulated Fast Switchable Adhesion within Glass Transition Zone of an Epoxy Polymer. Langmuir.

[30] Arena D., Verde-Sesto E., Pomposo J.A. (2022). Stars, combs and bottlebrushes of elastic single-chain nanoparticles. Polymer.

[31] Li Z., Liu J., Zhang Z., Gao Y., Liu L., Zhang L., Yuan B. (2018). Molecular dynamics simulation of the viscoelasticity of polymer nanocomposites under oscillatory shear: Effect of interfacial chemical coupling. RSC Adv..

[32] Wang X., Zhang Z., Zhang Y., Zhang G., Wang F. (2024). Study on association behavior and solution properties of poly (acrylic acid-alkyl polyoxyethylene acrylate) amphiphilic copolymers. Iran. Polym. J..

[33] (1989). Standard of the People’s Republic of China. Determination for Limiting Viscosity Number of Poly-Acrylamide.

[34] Tam K.C., Seng W.P., Jenkins R.D., Bassett D.R. (2000). Rheological and microcalorimetric studies of a model alkali-soluble associative polymer (HASE) in nonionic surfactant solutions. J. Polym. Sci. Part B Polym. Phys..

[35] Zhang H., Feng Y. (2021). Dependence of intrinsic viscosity and molecular size on molecular weight of partially hydrolyzed polyacrylamide. J. Appl. Polym. Sci..

[36] Nakamoto M., Kasai Y., Tanaka T., Yamamoto T. (2020). Measurement of liquid/liquid interfacial tension with water on liquid paraffin by the floating drop method. Colloids Surf. A Physicochem. Eng. Asp..

[37] Palominos M.A., Vilches D., Bossel E., Soto-Arriaza M.A. (2016). Interaction between amphipathic triblock copolymers and L-α-dipalmitoyl phosphatidylcholine large unilamellar vesicles. Colloids Surf. B Biointerfaces.

[38] Zastre J.A., Jackson J.K., Wong W., Burt H.M. (2008). P-Glycoprotein Efflux Inhibition by Amphiphilic Diblock Copolymers: Relationship between Copolymer Concentration and Substrate Hydrophobicity. Mol. Pharm..

[39] Di S., Ma S., Wei Y., Jing H. (2025). Study on the Mechanisms of Microscopic Remaining Oil Flow During Cyclic Water Flooding. ACS Omega.

[40] Ju Y., Xi C., Zheng J., Gong W., Wu J., Wang S., Mao L. (2022). Study on three-dimensional immiscible water-Oil two-phase displacement and trapping in deformed pore structures subjected to varying geostress via in situ computed tomography scanning and additively printed models. Int. J. Eng. Sci..

[41] Li X., Xiao K., Wang R., Li X. (2022). Experimental Research on Enhanced Oil Recovery Methods for Gas Injection of Fractured Reservoirs Based on Microfluidic Chips. ACS Omega.

[42] Wang X., Mao L., Kong X. (2025). Microscopic effects of long-term water injection on reservoir pore structure. Geoenergy Sci. Eng..

[43] Wang X., Wang F., Zhang Y., Ding X., Zhang G., Zhou T., Wang X., Zhang Z. (2024). Novel hydrophobic associative copolymers for natural gas hydrate fracturing fluids. J. Mol. Liq..

[44] Hiller W., Grabe B. (2023). The Universal Calibration for Structure- and Solvent-Independent Molar Mass Determinations of Polymers Using Diffusion-Ordered Spectroscopy. Anal. Chem..

[45] Štěpánek P., Tuzar Z., Kadlec P., Kříž J. (2007). A Dynamic Light Scattering Study of Fast Relaxations in Polymer Solutions. Macromolecules.

[46] Ohshima A., Sato T., Teramoto A. (2000). Optically Labeled Dynamic Light Scattering for Ternary Solutions Containing Two Polymer Species. Macromolecules.

[47] Cao H., Li Y., Gao W., Cao J., Sun B., Zhang J. (2023). Experimental investigation on the effect of interfacial properties of chemical flooding for enhanced heavy oil recovery. Colloids Surf. A Physicochem. Eng. Asp..

[48] Zhang R., Lu H., Wei L., Lin X., Yang Z., Qi J., Zhang Z. (2025). Self-Assembly at Oil-Water Interfaces Driven by Solubility Differences and Polar-Hydrophobic Interactions: An Insight into a Highly Mechanical Performance Gel with Gradients. Langmuir.

[49] Bian Z., Duan Z., An Q., Yang D., Liu Z. (2025). New insights of composite collector comprised of kerosene and a non-ionic surfactant for coal gangue flotation decarbonization: Experiment, characterization and molecular dynamics simulation. Chem. Eng. Res. Des..

[50] Li P., Zhang F., Gong Y., Tang J., Zhang C., Sun Z., Liu G., Li X. (2021). Synthesis and properties of functional polymer for heavy oil viscosity reduction. J. Mol. Liq..

[51] Jiang L., Xiang H., Xiaerbati, Xu J., Lv J., Lei H., Wei N., Song Y. (2025). Investigation of CO_2_ displacement oil with modified diffusion model in high water cut oil reservoir. Fuel.

[52] Bai M., Zhang Z., Yang E., Du S. (2025). Experimental study of microscopic oil production and CO_2_ storage in low-permeable reservoirs. Pet. Sci..

